# The consensus number of a cryptocurrency

**DOI:** 10.1007/s00446-021-00399-2

**Published:** 2021-10-23

**Authors:** Rachid Guerraoui, Petr Kuznetsov, Matteo Monti, Matej Pavlovic, Dragos-Adrian Seredinschi

**Affiliations:** 1 École Polytechnique Fédérale de Lausanne (EPFL), Lausanne, Switzerland; 2 LTCI, Télécom Paris, Institut Polytechnique de Paris, Paris, France

**Keywords:** Distributed computing, Cryptocurrency, Consensus, Blockchain, Decentralized payments

## Abstract

Many blockchain-based algorithms, such as Bitcoin, implement a decentralized asset transfer system, often referred to as a cryptocurrency. As stated in the original paper by Nakamoto, at the heart of these systems lies the problem of preventing double-spending; this is usually solved by achieving consensus on the order of transfers among the participants. In this paper, we treat the asset transfer problem as a concurrent object and determine its consensus number, showing that consensus is, in fact, not necessary to prevent double-spending. We first consider the problem as defined by Nakamoto, where only a single process—the account owner—can withdraw from each account. Safety and liveness need to be ensured for correct account owners, whereas misbehaving account owners might be unable to perform transfers. We show that the consensus number of an asset transfer object is 1. We then consider a more general *k*-shared asset transfer object where up to *k* processes can atomically withdraw from the same account, and show that this object has consensus number *k*. We establish our results in the context of shared memory with benign faults, allowing us to properly understand the level of difficulty of the asset transfer problem. We also translate these results in the message passing setting with Byzantine players, a model that is more relevant in practice. In this model, we describe an asynchronous Byzantine fault-tolerant asset transfer implementation that is both simpler and more efficient than state-of-the-art consensus-based solutions. Our results are applicable to both the permissioned (private) and permissionless (public) setting, as normally their differentiation is hidden by the abstractions on top of which our algorithms are based.

## Introduction

The Bitcoin protocol, introduced in 2008 by Satoshi Nakamoto, implements a *cryptocurrency*: an electronic decentralized asset transfer system [39]. Since then, many alternatives to Bitcoin came to prominence. These include major cryptocurrencies such as Ethereum [48] or Ripple [41], as well as systems sparked from research or industry efforts such as Bitcoin-NG [19], Algorand [24], ByzCoin [33], Stellar [38], Hyperledger Fabric [4], Corda [28], or Solida [2]. Each alternative brings novel approaches to implementing decentralized transfers, and sometimes offers a more general interface (known as smart contracts [44]) than the original protocol proposed by Nakamoto. They improve over Bitcoin in various aspects, such as performance, energy-efficiency, or security.

A common theme in these protocols, whether they are for basic transfers [34] or smart contracts [48], is that they seek to implement a *blockchain*—a distributed ledger where all the transfers in the system are totally ordered. Achieving total order among multiple inputs (e.g., transfers) is fundamentally a hard task, equivalent to solving *consensus* [27, 29]. Consensus [21], a central problem in distributed computing, is known for its notorious difficulty. It has no deterministic solution in asynchronous systems if just a single participant can fail [21]. Partially synchronous consensus algorithms are tricky to implement correctly [1, 12, 15] and face tough trade-offs between performance, security, and energy-efficiency [5, 8, 25, 47]. Not surprisingly, the consensus module is a major bottleneck in blockchain-based protocols [28, 43, 47].

A close look at Nakamoto’s original paper reveals that the central issue in implementing a decentralized asset transfer system (i.e., a cryptocurrency) is preventing *double-spending*, i.e., spending the same money more than once [39]. Bitcoin and numerous follow-up systems typically assume that total order—and thus consensus—is vital to preventing double-spending [22]. There seems to be a common belief, indeed, that a consensus algorithm is essential for implementing decentralized asset transfers [9, 25, 32, 39].

In this paper, we first show that consensus is not necessary to implement asset transfer in the shared memory model. We do so by casting the asset transfer problem as a *sequential object type* and determining that it has *consensus number* 1 in Herlihy’s hierarchy [29].^1^ We then use the insight provided by our result in the shared memory model—namely that processes do not need to establish total order on operations for asset transfer—to implement a fully asynchronous protocol that indeed implements asset transfer in the Byzantine message passing model without solving consensus.

The intuition behind this result is the following. An asset transfer object maintains a set of accounts. Each account is associated with an *owner* process that is the only one allowed to issue transfers withdrawing from this account. Every process can, however, read the balance of any account.

The main insight here is that relating accounts to unique owners obviates the need for consensus. It is the owner that decides on the order of transfers from its own account, without the need to agree with any other process—thus the consensus number 1. Other processes only validate the owner’s decisions, ensuring that causal relations across accounts are respected. We describe a simple asset transfer implementation using atomic-snapshot memory [3]. A withdrawal from an account is validated by relating the withdrawn amount with the incoming transfers found in the memory snapshot. Intuitively, as at most one withdrawal can be active on a given account at a time (as the account’s owner is a single sequential process), it is safe to declare the validated operation as successful and post it in the snapshot memory.

We also present a natural generalization of our result to the setting in which multiple processes are allowed to withdraw from the same account. A *k-shared* asset-transfer object allows up to *k* processes to execute outgoing transfers from the same account. We prove that such an object has consensus number *k* and thus allows for implementing *state machine replication* (now often referred to as *smart contracts*) among the *k* involved processes using *k*-consensus objects [31]. We show that *k*-shared asset transfer has consensus number *k* by reducing it to *k*-consensus (known to have consensus number *k*) and reducing *k*-consensus to asset transfer.

Studying the asset transfer problem in shared memory under crash faults provides crucial insight into the inherent synchronization complexity of the problem itself. Strictly speaking, however, results obtained in this model do not directly transfer to the (much more demanding) message passing model with Byzantine faults, under which most practical cryptocurrency systems operate.

Building on the intuitions from the shared memory model, we also present a practical solution to this problem in the setting of Byzantine fault-prone processes communicating via message passing. This setting matches realistic deployments of distributed systems. We describe an asset transfer implementation that does not resort to consensus. Instead, the implementation relies on a *secure broadcast* primitive that ensures uniform reliable delivery with only weak ordering guarantees [36, 37], circumventing hurdles imposed by consensus. In the *k*-shared case, our results imply that to execute some form of *smart contract* involving *k* users, consensus is only needed among these *k* nodes and not among all nodes in the system. In particular, should these *k* nodes be faulty, the rest of the accounts will not be affected.

To summarize, we argue that treating the asset transfer problem as a concurrent data structure and measuring its hardness through the lens of distributed computing helps to understand it and devise better solutions to it.

The rest of this paper is organized as follows. We first give the formal definition of the shared memory model and the asset transfer object type (Sect. 2). Then, we show that this object type has consensus number 1 (Sect. 3). Next, we generalize our result by proving that a *k*-shared asset transfer object has consensus number *k* (Sect. 4). Finally, we describe the implications of our results in the message passing model with Byzantine faults (Sects. 5 and 6) and discuss related work (Sect. 7).

## Shared memory model and asset-transfer object type

We now present the shared memory model (Sect. 2.1) and precisely define the problem of asset-transfer as a sequential object type (Sect. 2.2).

### Definitions

*Processes.* We assume a set $\varPi$ of *N* asynchronous processes that communicate by invoking atomic operations on shared memory objects. Processes are sequential—we assume that a process never invokes a new operation before obtaining a response from a previous one.

*Object types.* A sequential object type is defined as a tuple $T=(Q,q_0,O,R,\varDelta )$, where *Q* is a set of states, $q_0\in Q$ is an initial state, *O* is a set of operations, *R* is a set of responses and $\varDelta \subseteq Q\times \varPi \times O \times Q \times R$ is a relation that associates a state, a process identifier and an operation to a set of possible new states and corresponding responses. We assume that $\varDelta$ is total on the first three elements.

A *history* is a sequence of invocations and responses, each invocation or response associated with a process identifier. A *sequential history* is a history that starts with an invocation and in which every invocation is immediately followed with a response associated with the same process. A sequential history is *legal* if its invocations and responses respect the relation $\varDelta$ for some sequence of state assignments.

*Implementations.* An *implementation* of an object type *T* is a distributed algorithm that, for each process and invoked operation, prescribes the actions that the process needs to take to perform it. An *execution* of an implementation is a sequence of *events*: invocations and responses of operations or atomic accesses to shared abstractions. The sequence of events at every process must respect the algorithm assigned to it.

*Failures.* Processes are subject to *crash* failures (we consider more general Byzantine failures in Sect. 5). A process may halt prematurely, in which case we say that the process has *crashed*. A process is called *faulty* if it crashes during the execution. A process is *correct* if it is not faulty. All algorithms we present in the shared memory model are *wait-free*—every correct process eventually returns from each operation it invokes, regardless of an arbitrary number of other processes crashing or concurrently invoking operations.

*Linearizability.* For each pattern of operation invocations, the execution produces a *history*, i.e., a sequence of distinct invocations and responses, labelled with process identifiers and unique sequence numbers.

A projection of a history *H* to process *p*, denoted *H*|*p* is the subsequence of elements of *H* labelled with *p*. An invocation *o* by a process *p* is *incomplete* in *H* if it is not followed by a response in *H*|*p*. A history is *complete* if it has no incomplete invocations. A *completion* of *H* is a history ${\bar{H}}$ that is identical to *H* except that every incomplete invocation in *H* is either removed or *completed* by inserting a matching response somewhere after it.

An invocation $o_1$ *precedes* an invocation $o_2$ in *H*, denoted $o_1\prec _H o_2$, if $o_1$ is complete and the corresponding response $r_1$ precedes $o_2$ in *H*. Note that $\prec _H$ stipulates a partial order on invocations in *H*. A *linearizable* implementation (also said an *atomic object*) of type *T* ensures that for every history *H* it produces, there exists a completion ${\bar{H}}$ and a legal sequential history *S* such that (1) for all processes *p*, ${\bar{H}}|p=S|p$ and (2) $\prec _H\subseteq \prec _S$.

*Consensus number.* The problem of *consensus* consists for a set of processes to *propose* values and *decide* on the proposed values so that no two processes decide on different values and every correct process decides. The *consensus number* of a type *T* is the maximal number of processes that can solve consensus using atomic objects of type *T* and read-write registers.

### The asset transfer object type

Let ${\mathcal {A}}$ be a set of *accounts* and $\mu : {\mathcal {A}}\rightarrow 2^{\varPi }$ be an “owner” map that associates each account with a set of processes that are, intuitively, allowed to debit the account. We define the asset-transfer object type associated with ${\mathcal {A}}$ and $\mu$ as a tuple $(Q,q_0,O,R,\varDelta )$, where:

- The set of states *Q* is the set of all possible maps $q:\;{\mathcal {A}}\rightarrow \mathbb {N}$. Intuitively, each state of the object assigns each account its *balance*.
- The initialization map $q_0:\;{\mathcal {A}}\rightarrow \mathbb {N}$ assigns the initial balance to each account.
- Operations and responses of the type are defined as $O=\{\textit{transfer}(a,b,x):\; a,b\in {\mathcal {A}},\,x\in \mathbb {N}\}\cup \{\textit{read}(a):\;a\in {\mathcal {A}}\}$ and $R=\{\textit{true},\textit{false}\}\cup \mathbb {N}$.
- $\varDelta$ is the set of valid state transitions. For a state $q\in Q$, a process $p\in \varPi$, an operation $o\in O$, a response $r\in R$ and a new state $q'\in Q$, the tuple $(q,p,o,q',r)\in \varDelta$ if and only if one of the following conditions is satisfied:
  - $o=\textit{transfer}(a,b,x) \wedge p\in \mu (a)$ $\wedge$ $q(a)\ge x$ $\wedge$ $q'(a)=q(a)-x$ $\wedge$ $q'(b)=q(b)+x \wedge \forall c \in {{\mathcal {A}}}\setminus \{a, b\}: q'(c) = q(c)$ (all other accounts unchanged) $\wedge$ $r=\textit{true}$; In other words, operation $\textit{transfer}(a,b,x)$ invoked by process *p* *succeeds* if and only if *p* is the owner of the source account *a* and account *a* has enough balance, and if it does, *x* is transferred from *a* to the destination account *b*. We call a $\textit{transfer}(a,b,x)$ operation *outgoing* for *a* and *incoming* for *b*; respectively, the *x* units are called *outgoing* for *a* and *incoming* for *b*. The response being *true* we call the transfer *successful*.
  - $o=\textit{transfer}(a,b,x)$ $\wedge$ ($p\notin \mu (a)$ $\vee$ $q(a)< x$) $\wedge$ $q'=q$ $\wedge$ $r=\textit{false}$; A transfer *fails* (having *false* as response) without modifying the state ($q'=q$) if the invoking process is not the owner of account *a* or if *a* has insufficient balance.
  - $o=\textit{read}(a)$ $\wedge$ $q=q'$ $\wedge$ $r=q(a)$. Operation $\textit{read}(a)$ simply returns the balance of *a* and leaves the account balances untouched.

Our model of the asset-transfer object defines a static number of participants and their accounts. Initial balances are also part of the object definition. In practice, such a model is closer to private/permissioned blockchain systems than to public/permissionless ones. Bootstrapping and dynamic reconfiguration of the accounts and membership are out of the scope of this paper. We believe, however, that our model of asset transfer serves a solid base for extensions addressing these issues.

As in Nakamoto’s original paper [39], we assume for the moment that an asset-transfer object has at most one owner per account: $\forall a \in {\mathcal {A}}: |\mu (a)| \le 1$. Later we lift this assumption and consider more general *k*-shared asset-transfer objects with arbitrary owner maps $\mu$ (Sect. 4). For the sake of simplicity, we also restrict ourselves to transfers with a single source account and a single destination account. However, our definition (and implementation) of the asset-transfer object type can trivially be extended to support transfers with multiple source accounts (all owned by the same sequential process) and multiple destination accounts.

## Asset transfer has consensus number 1

In this section, we show that the asset-transfer type can be wait-free implemented using only an atomic snapshot object [3]. Since atomic snapshot has consensus number 1 (i.e., can be implemented on top of read-write registers in a shared memory system with crash failures) [29], it follows that the asset-transfer type also has consensus number 1.

**Fig. 1:**
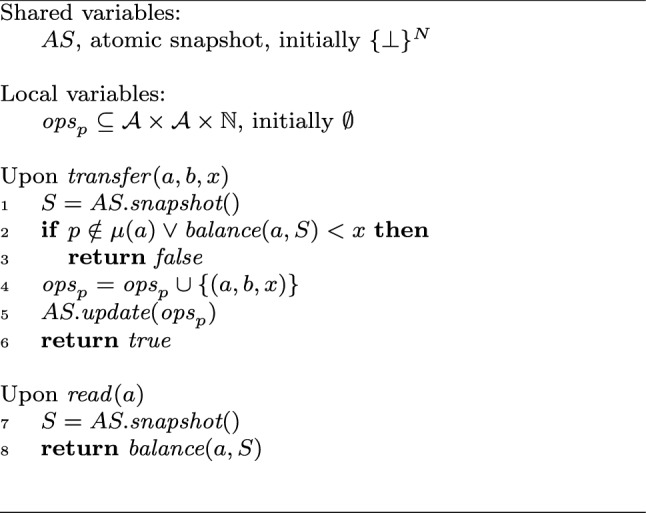
Wait-free implementation of asset-transfer: code for process *p*

Consider an asset-transfer object associated with a set of accounts ${\mathcal {A}}$ and an ownership map $\mu$ where $\forall a\in {\mathcal {A}}$, $|\mu (a)|\le 1$. Our implementation is described in Fig. 1. Every process *p* is associated with a distinct location in an atomic snapshot object [3] storing the set of all successful *transfer* operations executed by *p* so far. Since each account is owned by at most one process, all outgoing transfers for an account appear in a single location of the atomic snapshot (associated with the owner process).

Recall that the atomic snapshot (AS) memory is represented as a vector of *N* shared variables that can be accessed with two atomic operations: *update* and *snapshot*. An *update* operation modifies the value at a given position of the vector and a *snapshot* returns the state of the whole vector. We implement the *read* and *transfer* operations as follows.

- To read the balance of an account *a*, the process simply takes a snapshot *S* and returns the initial balance plus the sum of incoming amounts minus the sum of all outgoing amounts. We denote this number by $\textit{balance}(a,S)$. As we argue below, the result is guaranteed to be non-negative, i.e., the implementation is correct with respect to the type specification.
- To perform $\textit{transfer}(a,b,x)$, a process *p*, the owner of *a*, takes a snapshot *S* and computes $\textit{balance}(a,S)$. If the amount to be transferred does not exceed $\textit{balance}(a,S)$, we add the transfer operation to the set of *p*’s operations in the snapshot object via an *update* operation and return $\textit{true}$. Otherwise, the operation returns $\textit{false}$.

### Theorem 1

The asset-transfer object type has a wait-free implementation in the read-write shared memory model.

### Proof

Fix an execution *E* of the algorithm in Fig. 1. Atomic snapshots can be wait-free implemented in the read-write shared memory model [3]. As every operation only involves a finite number of atomic snapshot accesses, every process completes each of the operations it invokes in a finite number of its own steps.

Let $\textit{Ops}$ be the set of:

- All invocations of *transfer* or *read* in *E* that returned, and
- All invocations of *transfer* in *E* that completed the *update* operation (line 5).

Let *H* be the history of *E*. We define a completion of *H* and, for each $o\in \textit{Ops}$, we define a linearization point as follows:

- If *o* is a *read* operation, it linearizes at the linearization point of the *snapshot* operation in line 7.
- If *o* is a *transfer* operation that returns *false*, it linearizes at the linearization point of the *snapshot* operation in line 1.
- If *o* is a *transfer* operation that completed the *update* operation, it linearizes at the linearization point of the *update* operation in line 5. If *o* is incomplete in *H*, we complete it with response $\textit{true}$.

Let ${\bar{H}}$ be the resulting complete history and let *L* be the sequence of complete invocations of ${\bar{H}}$ in the order of their linearization points in *E*. Note that, by the way we linearize invocations, the linearization of a prefix of *E* is a prefix of *L*.

Now we show that *L* is legal and, thus, *H* is linearizable. We proceed by induction, starting with the empty (trivially legal) prefix of *L*. Let $L_{\ell }$ be the legal prefix of the first $\ell$ invocations and *op* be the $(\ell +1)$st operation of *L*. Let *op* be invoked by process *p*. The following cases are possible:

- *op* is a *read*(*a*): the snapshot taken at the linearization point of *op* contains all successful transfers concerning *a* in $L_{\ell }$. By the induction hypothesis, the resulting balance is non-negative.
- *op* is a failed *transfer*(*a*, *b*, *x*): the snapshot taken at the linearization point of *op* contains all successful transfers concerning *a* in $L_{\ell }$. By the induction hypothesis, the resulting balance is non-negative.
- *op* is a successful *transfer*(*a*, *b*, *x*): by the algorithm, before the linearization point of *op*, process *p* took a snapshot. Let $L_{k}$, $k\le \ell$, be the prefix of $L_{\ell }$ that only contain operations linearized before the point in time when the snapshot was taken by *p*. We observe that $L_k$ includes a *subset* of all incoming transfers on *a* and *all* outgoing transfers on *a* in $L_{\ell }$. Indeed, as *p* is the owner of *a* and only the owner of *a* can perform outgoing transfers on *a*, all outgoing transfers in $L_{\ell }$ were linearized before the moment *p* took the snapshot within *op*. Thus, $\textit{balance}(a,L_k) \le \textit{balance}(a,L_{\ell })$.^2^ By the algorithm, as $op={\textit{transfer}}(a,b,x)$ succeeds, we have $\textit{balance}(a,L_k)\ge x$. Thus, $\textit{balance}(a,L_{\ell })\ge x$ and the resulting balance in $L_{\ell +1}$ is non-negative.

Thus, *H* is linearizable. $\square$

### Corollary 1

The asset-transfer object type has consensus number 1.

## *k*-shared asset transfer has consensus number *k*

We now consider the case with an arbitrary owner map $\mu$. We show that an asset-transfer object’s consensus number is the maximal number of processes sharing an account. More precisely, the consensus number of an asset-transfer object is $\max _{a\in {\mathcal {A}}}|\mu (a)|$.

We say that an asset-transfer object, defined on a set of accounts ${\mathcal {A}}$ with an ownership map $\mu$, is *k-shared* iff $\max _{a\in {\mathcal {A}}}|\mu (a)|=k$. In other words, the object is *k*-shared if $\mu$ allows at least one account to be owned by *k* processes, and no account is owned by more than *k* processes.

We show that the consensus number of any *k*-shared asset-transfer object is *k*, which generalizes our result in Corollary 1. We first show that such an object has consensus number *at least k* by implementing consensus for *k* processes using only registers and an instance of *k*-shared asset-transfer. We then show that *k*-shared asset-transfer has consensus number *at most k* by reducing it to $k$-consensus, an object known to have consensus number *k* [31].

### Lemma 1

Consensus has a wait-free implementation for *k* processes in the read-write shared memory model equipped with a single *k*-shared asset-transfer object.

**Fig. 2:**
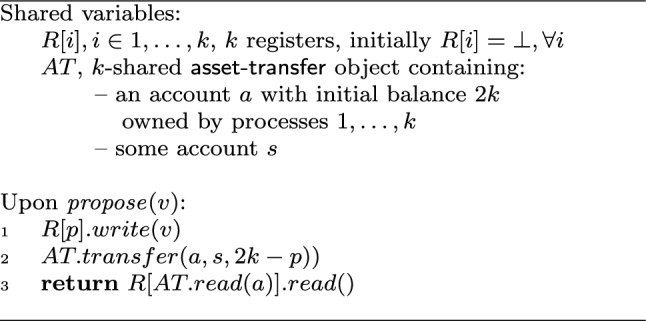
Wait-free implementation of consensus among *k* processes using a *k*-shared asset-transfer object. Code for process $p\in \{1,\ldots ,k\}$

### Proof

We now provide a wait-free algorithm that solves consensus among *k* processes using only registers and an instance of *k*-shared asset-transfer. The algorithm is described in Fig. 2. Intuitively, *k* processes use one shared account *a* to elect one of them whose input value will be decided. Before a process *p* accesses the shared account, *p* announces its input in a register (line 1). Process *p* then tries to perform a transfer from account *a* to another account. The amount withdrawn this way from account *a* is chosen specifically such that:

- only one transfer operation can ever succeed, and
- if the transfer succeeds, the remaining balance on *a* will uniquely identify process *p*.

To satisfy the above conditions, we initialize the balance of account *a* to 2*k* and have each process $p\in \{1,\ldots ,k\}$ transfer $2k-p$ (line 2). Note that transfer operations invoked by distinct processes $p,q\in \{1,\ldots ,k\}$ have arguments $2k-p$ and $2k-q$, and $2k-p+2k-q\ge 2k-k+2k-(k-1)=2k+1$. The initial balance of *a* is only 2*k* and no incoming transfers are ever executed. Therefore, the first transfer operation to be applied to the object succeeds (no transfer tries to withdraw more then 2*k*) and the remaining operations will have to fail due to insufficient balance.

When *p* reaches line 3, at least one transfer must have succeeded:

- either *p*’s transfer succeeded, or
- *p*’s transfer failed due to insufficient balance, in which case some other process must have previously succeeded.

Let *q* be the process whose transfer succeeded. Thus, the balance of account *a* is $2k - (2k - q) = q$. Since *q* performed a transfer operation, by the algorithm, *q* must have previously written its proposal to the register *R*[*q*]. Regardless of whether $p = q$ or $p \ne q$, reading the balance of account *a* returns *q* and *p* decides the value of *R*[*q*]. $\square$

To prove that *k*-shared asset-transfer has consensus number at most *k*, we reduce *k*-shared asset-transfer to $k$-consensus. A $k$-consensus object exports a single operation *propose* that, the first *k* times it is invoked, returns the argument of the first invocation. All subsequent invocations return $\bot$. Given that $k$-consensus is known to have consensus number exactly *k* [31], a wait-free algorithm implementing *k*-shared asset-transfer using only registers and $k$-consensus objects implies that the consensus number of *k*-shared asset-transfer is not more than *k*.

**Fig. 3:**
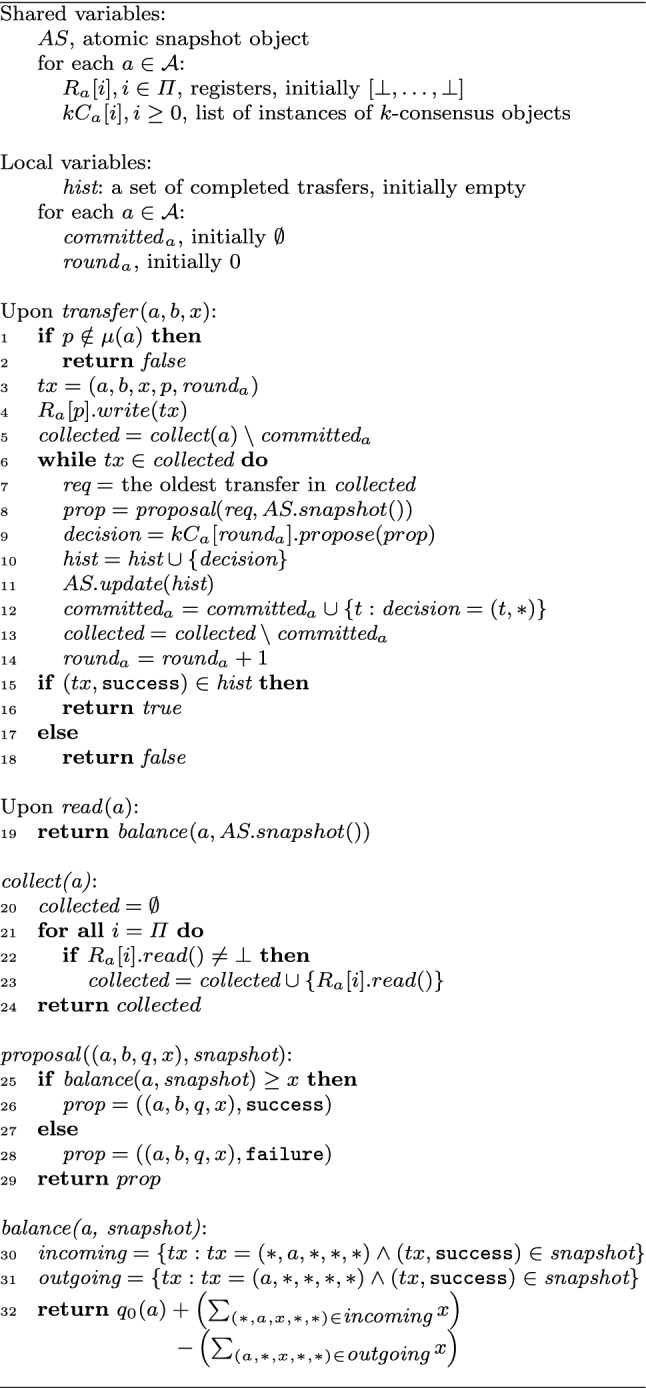
Wait-free implementation of a *k*-shared asset-transfer object using $k$-consensus objects. Code for process *p*

The algorithm reducing *k*-shared asset-transfer to $k$-consensus is given in Fig. 3. Before presenting a formal correctness argument, we first informally explain the intuition of the algorithm. In our reduction, we associate a series of $k$-consensus objects with every account *a*. Up to *k* owners of *a* use the $k$-consensus objects to agree on the order of outgoing transfers for *a*.

We maintain the state of the implemented *k*-shared asset-transfer object using an atomic snapshot object *AS*. Every process *p* uses a distinct entry of *AS* to store a set $\textit{hist}$. $\textit{hist}$ is a subset of all completed outgoing transfers from accounts that *p* owns (and thus is allowed to debit). For example, if *p* is the owner of accounts *d* and *e*, *p*’s $\textit{hist}$ contains outgoing transfers from *d* and *e*. Each element in the $\textit{hist}$ set is represented as $((a, b, x, s, r), \textit{result})$, where *a*, *b*, and *x* are the respective source account, destination account, and the amount transferred, *s* is the originator of the transfer, and *r* is the *round* in which the transfer was invoked by the originator. The value of $\textit{result} \in \{\texttt {success}, \texttt {failure}\}$ indicates whether the transfer succeeds or fails. A transfer becomes “visible” when any process inserts it in its corresponding entry of *AS*.

To read the balance of account *a*, a process takes a snapshot of *AS*, and then sums the initial balance $q_0(a)$ and amounts of all successful incoming transfers, and subtracts the amounts of successful outgoing transfers found in *AS*. We say that a successful transfer *tx* is in a snapshot *AS* (denoted by $(tx, \texttt {success}) \in AS$) if there exists an entry *e* in *AS* such that $(tx, \texttt {success}) \in AS[e]$.

To execute a transfer *o* outgoing from account *a*, a process *p* first announces *o* in a register $R_a$ that can be written by *p* and read by any other process. This enables a “helping” mechanism needed to ensure wait-freedom to the owners of *a* [29].

Next, *p* collects the transfers proposed by other owners and tries to agree on the order of the collected transfers and their results using a series of $k$-consensus objects. For each account, the agreement on the order of transfer-result pairs proceeds in rounds. Each round is associated with a $k$-consensus object which *p* invokes with a proposal chosen from the set of collected transfers. Since each process, in each round, only invokes the $k$-consensus object once, no $k$-consensus object is invoked more than *k* times and thus each invocation returns a value (and not $\bot$).

A transfer-result pair as a proposal for the next instance of $k$-consensus is chosen as follows. Process *p* picks the “oldest” collected but not yet committed operation (based on the round number $\textit{round}_a$ attached to the transfer operation when a process announces it; ties are broken using process IDs). Choosing the oldest operation ensures wait-freedom by preventing a process from indefinitely helping other processes, given only a finite number of possible older operations proposed by other processes.

Then, *p* takes a snapshot of *AS* and checks whether account *a* has sufficient balance according to the state represented by the snapshot, and equips the transfer with a corresponding success / failure flag. The resulting transfer-result pair constitutes *p*’s proposal for the next instance of $k$-consensus. The currently executed transfer by process *p* returns as soon as it is decided by a $k$-consensus object, the flag of the decided value (*success*/*failure*) indicating the transfer’s response (*true*/*false*).

### Lemma 2

The *k*-shared asset-transfer object type has a wait-free implementation in the read-write shared memory model equipped with *k*-consensus objects.

### Proof

We essentially follow the footpath of the proof of Theorem 1. Fix an execution *E* of the algorithm in Fig. 3. Let *H* be the history of *E*.

To perform a transfer *o* on an account *a*, *p* *registers* it in $R_a[p]$ (line 4) and then proceeds through a series of $k$-consensus objects, each time collecting $R_{a}$ to learn about the transfers concurrently proposed by other owners of *a*. Recall that each $k$-consensus object is wait-free. Suppose, by contradiction, that *o* is registered in $R_{a}$ but is never decided by any instance of $k$-consensus. Eventually, however, *o* becomes the request with the lowest round number in $R_{a}$ and, thus, some instance of $k$-consensus will be only accessed with *o* as a proposed value (line 9). By validity of $k$-consensus, this instance will return *o* and, thus, *p* will be able to complete *o*.

Let $\textit{Ops}$ be the set of all complete operations and all *transfer* operations *o* such that some process completed the update operation (line 11) in *E* with an argument including *o* (the atomic snapshot and $k$-consensus operation has been linearized). Intuitively, we include in $\textit{Ops}$ all operations that *took effect*, either by returning a response to the user or by affecting other operations. Recall that every such *transfer* operation was agreed upon in an instance of $k$-consensus, let it be $kC^{o}$. Therefore, for every such *transfer* operation *o*, we can identify the process $q^{o}$ whose proposal has been decided in that instance.

We now determine a completion of *H* and, for each $o\in \textit{Ops}$, we define a linearization point as follows:

- If *o* is a *read* operation, it linearizes at the linearization point of the snapshot operation (line 19).
- If *o* is a *transfer* operation that returns *false*, it linearizes at the linearization point of the snapshot operation (line 8) performed by $q^{o}$ just before it invoked $kC^{o}.\textit{propose}()$.
- If *o* is a *transfer* operation that some process included in the update operation (line 11), it linearizes at the linearization point of the *first* update operation in *H* (line 11) that includes *o*. Furthermore, if *o* is incomplete in *H*, we complete it with response $q^{o}$ determined before proposing *o* (line 8).

Let ${\bar{H}}$ be the resulting complete history and let *L* be the sequence of complete operations of ${\bar{H}}$ in the order of their linearization points in *E*. Note that, by the way we linearize operations, the linearization of a prefix of *E* is a prefix of *L*. Also, by construction, the linearization point of an operation belongs to its interval.

Now we show that *L* is legal and, thus, *H* is linearizable. We proceed by induction, starting with the empty (trivially legal) prefix of *L*. Let $L_{\ell }$ be the legal prefix of the first $\ell$ operation and *op* be the $(\ell +1)$st operation of *L*. Let *op* be invoked by process *p*. The following cases are possible:

- *op* is a *read*(*a*): the snapshot taken at *op*’s linearization point contains all successful transfers concerning *a* in $L_{\ell }$. By the induction hypothesis, the resulting balance is non-negative.
- *op* is a failed *transfer*(*a*, *b*, *x*): the snapshot taken at the linearization point of *op* contains all successful transfers concerning *a* in $L_{\ell }$. By the induction hypothesis, the balance corresponding to this snapshot non-negative. By the algorithm, the balance is less than *x*.
- *op* is a successful *transfer*(*a*, *b*, *x*). Let $L_{s}$, $s\le \ell$, be the prefix of $L_{\ell }$ that only contains operations linearized before the moment of time when $q^{o}$ has taken the snapshot just before accessing $kC^{o}$. As before accessing $kC^{o}$, *q* went through all preceding $k$-consensus objects associated with *a* and put the decided values in *AS*, $L_{s}$ must include *all* outgoing *transfer* operations for *a*. Furthermore, $L_s$ includes a *subset* of all incoming transfers on *a*. Thus, $\textit{balance}(a,L_k) \le \textit{balance}(a,L_{\ell })$. By the algorithm, as $op={\textit{transfer}}(a,b,x)$ succeeds, we have $\textit{balance}(a,L_k)\ge x$. Thus, $\textit{balance}(a,L_{\ell })\ge x$ and the resulting balance in $L_{\ell +1}$ is non-negative.

Thus, *H* is linearizable. $\square$

### Theorem 2

A *k*-shared asset-transfer object has consensus number *k*.

### Proof

It follows directly from Lemma 1 that *k*-shared asset-transfer has consensus number at least *k*. Moreover, it follows from Lemma 2 that *k*-shared asset-transfer has consensus number at most *k*. Thus, the consensus number of *k*-shared asset-transfer is exactly *k*. $\square$

It is worth noting that Theorem 2 implies that, in a more demanding model than shared memory with crash faults (particularly the Byzantine message passing model), solving consensus among *k* processes is necessary, but not necessarily sufficient for implementing *k*-shared asset transfer.

## Asset transfer in message passing

We established our theoretical results in a shared memory system with crash failures, proving that consensus is not necessary for implementing an asset transfer system. Moreover, a natural generalization of such a system where up to *k* processes have access to atomic operations on the same account has consensus number *k*. These results help us understand the level of difficulty of certain problems in the domain of cryptocurrencies. While suggesting that agreement might be unnecessary in the Byzantine message passing model (on which most blockchain systems are based) as well, they do not prove it. To show that agreement is indeed not needed for blockchain systems, we need an algorithm for the Byzantine message passing model, i.e., one where processes (some of which are potentially malicious) communicate by exchanging messages.

In this section we present such an extension of our results to the message passing system with Byzantine failures. Instead of consensus, we rely on a *secure broadcast* primitive that provides reliable delivery with weak (weaker than FIFO) ordering guarantees [37]. Using secure broadcast, processes announce their transfers to the rest of the system. We establish *dependencies* among these transfers that induce a partial order. Using a method similar to (a weak form of) vector clocks [20], we make sure that each process applies the transfers respecting this dependency-induced partial order. In a nutshell, a transfer only depends on all previous transfers outgoing from the same account, and on a subset of transfers incoming to that account. Each transfer operation corresponds to one invocation of secure broadcast by the corresponding account’s owner. The message being broadcast carries, in addition to the transfer itself, references to the transfer’s dependencies.

As secure broadcast only provides liveness if the sender is correct, faulty processes might not be able to perform any transfers. However, due to secure broadcast’s delivery properties, the correct processes will always have a consistent view of the system state.

Every transfer operation only entails a single invocation of secure broadcast and our algorithm does not send any additional messages. Our algorithm inherits the complexity from the underlying secure broadcast implementation, and there are plenty of such algorithms optimizing complexity metrics for various settings [10, 11, 23, 27, 36, 37, 46]. In practice, as shown by our implementation and evaluation [16], our solution outperforms a consensus-based one by 5*x* in throughput and while maintaining a sub-second latency.

The implementation can be further extended to solve the *k-shared* asset transfer problem. As we showed in Sect. 4, agreement among a subset of the processes is necessary in such a case. We associate each account (owned by up to *k* processes) with a Byzantine-fault tolerant state machine replication (BFT SMR) service executed by the owners [13] of that account. The BFT service assigns sequence numbers to transfers which the processes then submit to an extended version of the above-mentioned transfer protocol. As long as the replicated state machine is safe and live, we guarantee that every invoked transfer operation eventually returns. If an account becomes compromised (i.e., the safety or liveness of the BFT SMR is violated), only the corresponding account might lose liveness. In other words, outgoing transfers from the compromised account may not return, while safety and liveness of transfers from “healthy” accounts are always guaranteed. We describe this extension in more details later (Sect. 6).

In the rest of this section, we give details on the Byzantine message passing model, adapt our asset-transfer object accordingly (Sect. 5.1) and present its broadcast-based implementation (Sect. 5.2).

### Byzantine message passing model

A process is Byzantine if it deviates from the algorithm it is assigned, either by halting prematurely, in which case we say that the process is *crashed*, or performing actions that are not prescribed by its algorithm, in which case we say that the process is *malicious*. Malicious processes can perform arbitrary actions, except for ones that involve subverting cryptographic primitives (e.g. inverting secure hash functions). A process is called *faulty* if it is either crashed or malicious. A process is *correct* if it is not faulty and *benign* if it is not malicious. Note that every correct process is benign, but not necessarily vice versa.

We only require that the transfer system behaves correctly towards *benign* processes, regardless of the behavior of Byzantine ones. Informally, we want to require that no benign process can be a victim of a double-spending attack, i.e., every execution appears to benign processes as a correct sequential execution, respecting the original execution’s real-time ordering [29].

For the sake of efficiency, in our algorithm, we slightly relax the last requirement—while still preventing double-spending. We require that *successful* *transfer* operations invoked by benign processes constitute a legal sequential history that preserves the real-time order. A $\textit{read}$ or a failed $\textit{transfer}$ operation invoked by a benign process *p* can be “outdated”—it can be based on a stale state of *p*’s balance. Informally, one can view the system requirements as *linearizability* [30] for successful transfers and *sequential consistency* [6] for failed transfers and reads. As progress (liveness) guarantees, we require that every operation invoked by a correct process eventually completes.

In a nutshell, sequential consistency resembles linearizability with real-time constraints removed. Each process observes a sequence of events consistent with the sequential specification, but the effects of other processes’ invocations need not respect real-time order with respect to the process’ own invocations. One can argue that this relaxation incurs little impact on the system’s utility, since all incoming transfers are eventually applied. We discuss a fully linearizable implementation at the end of this section.

#### Definition 1

Let *E* be any execution of an implementation and *H* be the corresponding history. Let $\textit{ops}(H)$ denote the set of operations in *H* that were executed by correct processes in *E*. An asset-transfer object in message passing guarantees that each invocation issued by a correct process is followed by a matching response in *H*, and that there exists ${\bar{H}}$, a completion of *H*, such that:

1. Let ${\bar{H}}^{t}$ denote the sub-history of successful transfers of ${\bar{H}}$ performed by correct processes and $\prec _{{\bar{H}}}^t$ be the subset of $\prec _{{\bar{H}}}$ restricted to operations in ${\bar{H}}^{t}$. Then there exists a legal sequential history *S* such that (a) for every benign process *p*, ${\bar{H}}^{t}|p=S|p$ and (b) $\prec _{\bar{H}}^t\subseteq \prec _S$.
2. For every benign process *p*, there exists a legal sequential history $S_p$ such that:
  $$\textit{ops}({\bar{H}}) \subseteq \textit{ops}(S_p)$$, and
  $$S_p|p= {\bar{H}} |p$$.

Notice that property (2) implies that every update in *H* that affects the account of a correct process *p* is eventually included in *p*’s “local” history and, therefore, will reflect reads and transfer operations subsequently performed by *p*.

### Asset transfer implementation in message passing

Instead of consensus, we rely on a secure broadcast primitive that is strictly weaker than consensus and has a fully asynchronous implementation. It provides uniform reliable delivery despite Byzantine faults and so-called *source order* among delivered messages. The source order property, being weaker than FIFO, guarantees that messages from the same source are delivered in the same order by all correct processes. More precisely, the secure broadcast primitive we use in our implementation has the following properties [37]:

- **Integrity:** A benign process delivers a message *m* from a process *p* at most once and, if *p* is benign, only if *p* previously broadcast *m*.
- **Agreement:** If processes *p* and *q* are correct and *p* delivers *m*, then *q* delivers *m*.
- **Validity:** If a correct process *p* broadcasts *m*, then *p* delivers *m*.
- **Source order:** If *p* and *q* are benign and both deliver *m* from *r* and $m'$ from *r*, then they do so in the same order.

*Operation.* To perform a transfer *tx*, a process *p* securely broadcasts a message with the transfer details: the arguments of the $\textit{transfer}$ operation (see  Sect. 2.2) and some metadata. The metadata includes a per-process *sequence number* of *tx* and references to the *dependencies* of *tx*. The dependencies are transfers incoming to *p* that must be known to any process before applying *tx*. These dependencies impose a causal relation between transfers that must be respected when transfers are being applied. For example, suppose that process *p* makes a transfer *tx* to process *q* and *q*, after observing *tx*, performs another transfer $tx'$ to process *r*. *q*’s broadcast message will contain $tx'$, a local sequence number, and a reference to *tx*. Any process (not only *r*) will only evaluate the validity of $tx'$ after having applied *tx*. This approach is similar to using vector clocks for implementing causal order among events [20].

To ensure the authenticity of operations—so that no process is able to debit another process’s account—we assume that processes sign all their messages before broadcasting them. In practice, similar to Bitcoin and other transfer systems, every process possesses a public-private key pair that allows only *p* to securely initiate transfers from its corresponding account. For simplicity of presentation, we omit this mechanism in the algorithm pseudocode.

**Fig. 4:**
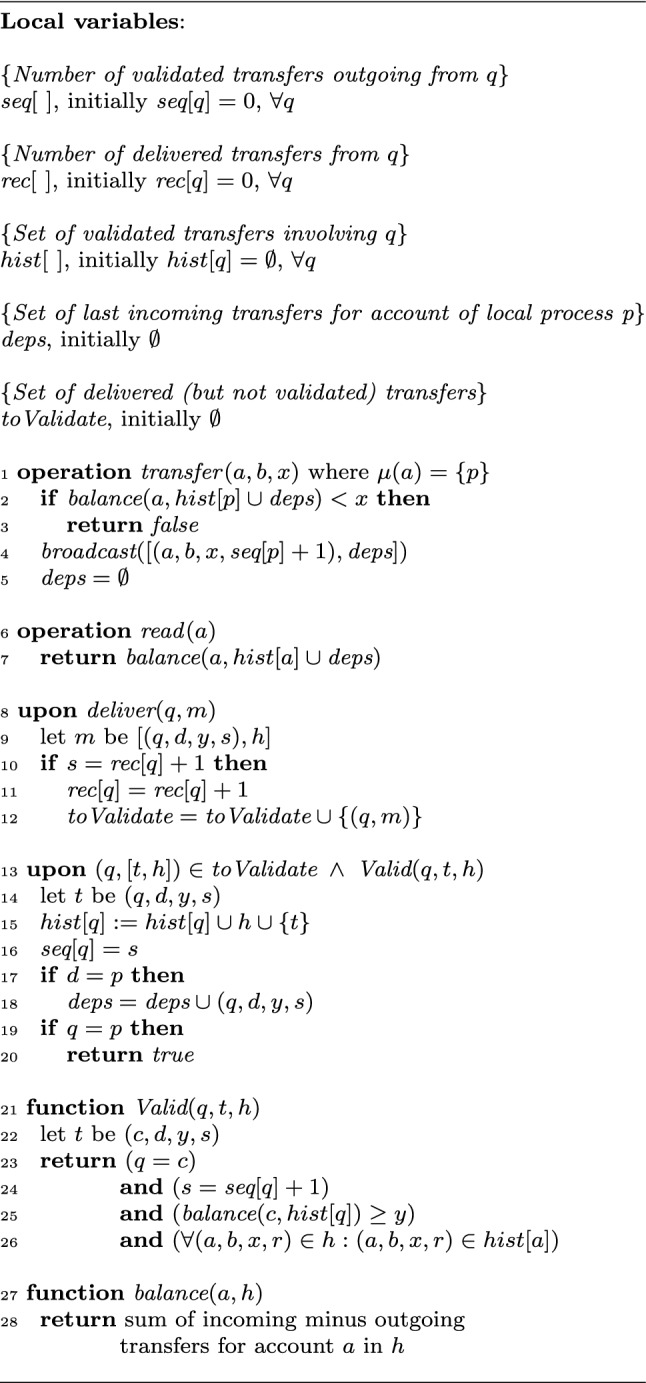
Consensusless transfer system based on secure broadcast. Code for every process *p*

Figure 4 describes the full algorithm implementing asset-transfer in a Byzantine-prone message passing system. Each process *p* maintains, for each process *q*, an integer $\textit{seq}[q]$ reflecting the number of transfers which process *q* initiated and which process *p* has validated and applied. Process *p* also maintains, for every process *q*, an integer $\textit{rec}[q]$ reflecting the number of transfers process *q* has initiated and process *p* has delivered (but not necessarily applied).

Additionally, there is also a list $\textit{hist}[q]$ of transfers which *involve* process *q*. We say that a transfer operation involves a process *q* if that transfer is either outgoing or incoming on the account of *q*. Each process *p* maintains as well a local variable $\textit{deps}$. This is a set of transfers *incoming* for *p* that *p* has applied since the last successful *outgoing* transfer—and thus is cleared after each invocation of *broadcast* (line 5).^3^ Finally, the set $\textit{toValidate}$ contains delivered transfers that are pending validation (i.e., have been delivered, but not yet validated).

To perform a $\textit{transfer}$ operation, process *p* first checks the balance of its own account, and if the balance is insufficient, returns $\textit{false}$ (line 3). Otherwise, process *p* broadcasts a message with this operation via the secure broadcast primitive (line 4). This message includes the three basic arguments of a $\textit{transfer}$ operation as well as $\textit{seq}[p]+1$ and dependencies $\textit{deps}$. Each correct process in the system eventually delivers this message via secure broadcast (line 8). Note that, given the assumption of no process executing more than one concurrent transfer, every process waits for delivery of its own message before initiating another broadcast. This effectively turns the source order property of secure broadcast into FIFO order. Upon delivery, process *p* checks this message for well-formedness (lines 9 and 10), and then adds it to the set of messages pending validation. We explain the validation procedure later.

Once a transfer passes validation (the predicate in line 13 is satisfied), process *p* applies this transfer on the local state. Applying a transfer means that process *p* adds this transfer and its dependencies to the history of the outgoing (line 15) account. If the transfer is incoming for local process *p*, it is also added to $\textit{deps}$, the set of current dependencies for *p* (line 18). If the transfer is outgoing for *p*, i.e., it is the currently pending $\textit{transfer}$ operation invoked by *p*, then the response $\textit{true}$ is returned (line 20).

To perform a *read*(*a*) operation for account *a*, process *p* simply computes the balance of this account based on the local history *hist*[*a*] (line 28).

*Validation.* Before applying a transfer *op* from some process *q*, process *p* validates *op* via the *Valid* function (lines 21–26). To be valid, *op* must satisfy four conditions. The first condition is that process *q* (the issuer of transfer *op*) must be the owner of the outgoing account for *op* (line 23). Second, any preceding transfers that process *q* issued must have been validated (line 24). Third, the balance of account *q* must not drop below zero (line 25). Finally, the reported dependencies of *op* (encoded in *h* of line 26) must have been validated and exist in $\textit{hist}[q]$.

#### Lemma 3

In any infinite execution of the algorithm (Fig. 4), every operation performed by a correct process eventually completes.

#### Proof

A transfer operation that fails or a read operation invoked by a correct process returns immediately (lines 3 and 7, respectively).

Consider a transfer operation *T* invoked by a correct process *p* that *succeeds* (i.e., passes the check in line 2), so *p* broadcasts a message with the transfer details using secure broadcast (line 4). By the validity property of secure broadcast, *p* eventually delivers the message (via the secure broadcast callback, line 8) and adds it to the $\textit{toValidate}$ set. By the algorithm, this message includes a set $\textit{deps}$ of operations (called *h*, line 9) that involve *p*’s account. This set includes transfers that process *p* delivered and validated after issuing the prior successful outgoing transfer (or since system initialization if there is no such transfer) but before issuing *T* (lines 4 and 5).

As process *p* is correct, it operates on its own account, respects the sequence numbers, and issues a transfer only if it has enough balance on the account. Thus, when it is delivered by *p*, *T* must satisfy the first three conditions of the $\textit{Valid}$ predicate (lines 23–25). Moreover, by the algorithm, all dependencies (labeled *h* in function $\textit{Valid}$) included in *T* are in the history *hist*[*p*] and, thus the fourth validation condition (line 26) also holds.

Thus, *p* eventually validates *T* and completes the operation by returning $\textit{true}$ in line 20. $\square$

#### Theorem 3

The algorithm in Fig. 4 implements an asset-transfer object type.

#### Proof

Fix an execution *E* of the algorithm, let *H* be the corresponding history.

Let ${\mathcal {V}}$ denote the set of all messages that were delivered (line 8) and validated (line 23) at correct processes in *E*. Every message $m=[(q,d,y,s),h]\in {\mathcal {V}}$ is put in *hist*[*q*] (line 15). We define an order $\preceq \subseteq {\mathcal {V}}\times {\mathcal {V}}$ as follows. For $m=[(q,d,y,s),h]\in {\mathcal {V}}$ and $m'=[(r,d',y',s'),h']\in {\mathcal {V}}$, we have $m\preceq m'$ if and only if one of the following conditions holds:

- $$q=r$$ and $$s<s'$$,
- $$(r,d',y',s')\in h$$, or
- there exists $m''\in {\mathcal {V}}$ such that $m\preceq m''$ and $m''\preceq m'$.

By the source order property of secure broadcast (see  Sect. 5.2), correct processes *p* and *r* deliver messages from any process *q* in the same order. By the algorithm in Fig. 4, a message from *q* with a sequence number *i* is added by a correct process to $\textit{toValidate}$ set only if the previous message from *q* added to $\textit{toValidate}$ had sequence number $i-1$ (line 10). Furthermore, a message $m=[(q,d,y,s),h]$ is validated at a correct process only if all messages in *h* have been previously validated (line 26). Therefore, $\preceq$ is acyclic and thus can be extended to a total order.

Let *S* be the sequential history constructed from any such total order on messages in ${\mathcal {V}}$ in which every message $m=[(q,d,y,s),h]$ is replaced with the invocation-response pair $\textit{transfer}(q,d,y);\textit{true}$.

By construction, every operation $\textit{transfer}(q,d,y)$ in *S* is preceded by a sequence of transfers that ensure that the balance of *q* does not drop below *y* (line 25). In particular, *S* includes all outgoing transfers from the account of *q* performed previously by *q* itself. Additionally *S* may order some *incoming* transfer to *q* that did not appear at *hist*[*q*] before the corresponding (*q*, *d*, *y*, *s*) has been added to it. But these “unaccounted” operations may only increase the balance of *q* and, thus, it is indeed legal to return $\textit{true}$.

By construction, for each correct process *p*, *S* respects the order of successful transfers issued by *p*. Thus, the subsequence of successful transfers in *H* “looks” linearizable to the correct processes: *H*, restricted to successful transfers witnessed by the correct processes, is consistent with a legal sequential history *S*.

Let *p* be a correct process in *E*. Now let ${\mathcal {V}}_p$ denote the set of all messages that were delivered (line 8) and validated (line 23) at *p* in *E*. Let $\preceq _p$ be the subset of $\preceq$ restricted to the elements in ${\mathcal {V}}_p$. Obviously, $\preceq _p$ is cycle-free and we can again extend it to a total order. Let $S_p$ be the sequential history build in the same way as *S* above. Similarly, we can see that $S_p$ is legal and, by construction, consistent with the local history of *all* operations of *p* (including reads and failed transfers).

By Lemma 3, every operation invoked by a correct process eventually completes. Thus, *E* indeed satisfies the properties of an asset-transfer object type. $\square$

### Linearizability

As stated above, our implementation provides linearizability for successful transfers and sequential consistency for reads and failed transfers. The fundamental reason for this is that the balance operation only consults local state without synchronizing with other processes.

What prevents the implementation from being fully linearizable is the following situation. A process *p* returns from a successful *transfer* operation *o* after having delivered its own broadcast message *m*. Then, some other process *q* invokes an operation $o'$ that queries *p*’s balance before *q* delivers *m*. Thus, even though *q* invokes $o'$ after *o* returned, the result of *q*’s operation does not reflect the effect of *o*, violating linearizability.

Intuitively, to achieve linearizability, the above algorithm needs to be extended by an acknowledgment mechanism that delays returning from an operation until the state observed/produced by the operation is guaranteed to be visible by other processes. In a practical setting, this has the disadvantage of adding extra communication delays.

## *k*-shared asset transfer in message passing

Our message-passing asset-transfer implementation can be naturally extended to the *k*-shared case, when some accounts are owned by up to *k* processes. Functionally, *k*-shared accounts are similar to “multi-sig” accounts used in Bitcoin and other blockchain systems, where a transfer must be signed by 1 out of *k* account owners. The concept of multi-sig accounts, however, only extends an application-level definition of what constitutes a valid transfer and has no implications on its ordering with respect to other transfers. This section provides an informal insight in how *k*-shared could be implemented in a Byzantine message passing model.

*k-shared BFT SMR service.* As we showed in Sect. 4, a purely asynchronous implementation of a *k*-shared asset-transfer does not exist, even in the benign shared-memory environment. To circumvent this impossibility, we assume that every account is associated with a Byzantine fault-tolerant state-machine replication (SMR) service (e.g., PBFT [13]) that is used by the account’s owners to order their outgoing transfers. In a nutshell, the function of an SMR service is to receive inputs from clients, establish a total order on those inputs and compute functions of the resulting sequence that it returns to clients. In particular, the account owners submit their issued transfers to the service, which assigns monotonically increasing *sequence numbers* to those transfers.

The service itself can be implemented by the owners themselves, acting both as *clients*, submitting requests, and *replicas*, reaching agreement on the order in which the requests must be served. As long as more than two thirds of the owners are correct, the service is *safe*, in particular, no sequence number is assigned to more than one transfer. Moreover, under the condition that the owners can eventually communicate within a bounded message delay, every request submitted by a correct owner is guaranteed to be eventually assigned a sequence number [13]. One can argue that it is much more likely that this assumption of *eventual synchrony* holds for a bounded set of owners, rather than for the whole set of system participants. Furthermore, communication complexity of such an implementation is polynomial in *k* and not in *N*, the number of processes.

*Account order in secure broadcast.* Consider even the case where the threshold of one third of Byzantine owners is exceeded, where the account may become blocked or, even worse, compromised. In this case, different owners may be able to issue two different transfers associated with the same sequence number.

This issue can be mitigated by a slight modification of the classical secure broadcast algorithm [37]. In addition to the properties of Integrity, Validity and Agreement of secure broadcast, the modified algorithm can implement the property of *account order*, generalizing the *source order* property (Sect. 5.2). Assume that each broadcast message is equipped with a sequence number (generated by the BFT service, as we will see below).

- **Account order:** If a benign process *p* delivers messages *m* (with sequence number *s*) and $m'$ (with sequence number $s'$) such that *m* and $m'$ are associated with the same account and $s<s'$, then *p* delivers *m* before $m'$.

Informally, the implementation works as follows. The sender sends the message (containing the account reference and the sequence number) it wants to broadcast to all and waits until it receives acknowledgements from a *quorum* of more than two thirds of the processes. A message with a sequence number *s* associated with an account *a* is only acknowledged by a benign process if the last message associated with *a* it delivered had sequence number $s-1$. Once a quorum is collected, the sender sends the message equipped with the signed quorum to all and delivers the message. This way, the benign processes deliver the messages associated with the same account in the same order. If the owners of an account send conflicting messages for the same sequence number, the account may block. However, and most importantly, even a compromised account is always prevented from double spending. Liveness of operations on a compromised account is not guaranteed, but safety and liveness of other operations remains unaffected.

*Putting it all together.* The resulting *k*-shared asset transfer system is a composition of a collection of BFT services (one per account), the modified secure broadcast protocol (providing the account-order property), and a slightly modified protocol in Fig. 4.

To issue a transfer operation *t* on an account *a* it owns, a process *p* first submits *t* to the associated BFT service to get a sequence number. Assuming that the account is not compromised and the service is consistent, the transfer receives a unique sequence number *s*. Note that the decided tuple (*a*, *t*, *s*) should be signed by a quorum of owners: this will be used by the other processes in the system to ensure that the sequence number has been indeed agreed upon by the owners of *a*. The process executes the protocol in Fig. 4, with the only modification that the sequence number *seq* is now not computed locally but adopted from the BFT service.

Intuitively, as the transfers associated with a given account are processed by the benign processes in the same order, the resulting protocol ensures that the history of successful transfers is linearizable. On the liveness side, the protocol ensures that every transfer on a non-compromised account is guaranteed to complete.

## Related work

Many systems address the problem of asset transfers, be they for a permissioned (private, with a trusted external access control mechanism) [4, 28, 32] or permissionless (public, prone to Sybil attacks) setting [2, 17, 24, 34, 39, 45]. Decentralized systems for the public setting are open to the world. To prevent malicious parties from overtaking the system, these systems rely on Sybil-proof techniques, e.g., proof-of-work [39], or proof-of-stake [7]. The above-mentioned solutions, whether for the permissionless or the permissioned environment, seek to solve consensus. They must inevitably rely on synchrony assumptions or randomization. Avalanche [45], for example, relaxes consensus by allowing an explicit (small) failure probability. By sidestepping consensus, we can provide a deterministic and asynchronous implementation.

It is worth noting that many of those solutions allow for more than just transfers, and support richer operations on the system state—so-called smart contracts. Our paper focuses on the original asset transfer problem, as defined by Nakamoto [39], and we do not address smart contracts, for certain forms of which consensus is indeed necessary. However, our approach allows for arbitrary operations, if those operations affect groups of the participants that can solve consensus among themselves. Potential safety or liveness violations of those operations (in case this group gets compromised) are confined to the group and do not affect the rest of the system.

In the blockchain ecosystem, a lot of work has been devoted to avoid a totally ordered chain of transfers. The idea is to replace the totally ordered linear structure of a blockchain with that of a directed acyclic graph (DAG) for structuring the transfers in the system. Notable systems in this spirit include Byteball [14], Vegvisir [32], Corda [28], Nano [35], or the GHOST protocol [42]. Even if these systems use a DAG to replace the classic blockchain, they still employ consensus.

We can also use a DAG to characterize the relation between transfers, but we do not resort to solving consensus to build the DAG, nor do we use the DAG to solve consensus. More precisely, we can regard each account as having an individual history. Each such history is managed by the corresponding account owner without depending on a global view of the system. Histories are loosely coupled through a causality relation established by dependencies among transfers.

The important insight that an asynchronous broadcast-style abstraction suffices for transfers appears in the literature as early as 2002, due to Pedone and Schiper [40]. Duan et. al. [18] introduce efficient Byzantine fault-tolerant protocols for storage and also build on this insight. So does recent work by Gupta [26] on financial transfers which seems closest to ours; the proposed algorithm is based on similar principles as some implementations of secure broadcast [36, 37]. To the best of our knowledge, however, we are the first to formally define the asset transfer problem as a shared object type, study its consensus number, and propose algorithms building on top of standard abstractions that are amenable to a real deployment in cryptocurrencies.

## Footnotes

1 The consensus number of an object type is the maximal number of processes that can solve consensus using only read-write shared memory and arbitrarily many objects of this type.

2 Analogously to $\textit{balance}(a,S)$ that computes the balance for account *a* based on the transfers contained in snapshot *S*, $\textit{balance}(a,L)$, if *L* is a sequence of operations, computes the balance of account *a* based on all transfers in *L*.

3 The algorithm would stay correct even without ever clearing *deps*. Clearing it, however, avoids broadcasting ever-growing messages.
